# Supplementary material for: Intergenerational epigenetic inheritance of cancer susceptibility in mammals
Source: eLife. 2019 Apr 9;8:e39380. doi: 10.7554/eLife.39380 (PMC6456297; doi:10.7554/eLife.39380)
Supplement: Figure 2—source data 2. — Counts represent total tumors including multiple tumors per mouse. Tumor rate (tumors/mouse) in parentheses. Kdm6a F2.controlA mice generated from a Cre-only sire; Kdm6a F2.controlB mice generated from a Kdm6a(fl)-only sire. [file elife-39380-fig2-data2.docx]

| **Tumor category** | **Control F1**  **(25 mice)** | **Utx F1**  **(22 mice)** | **Utx F2.cKO**  **(40 mice)** | **Utx F2.controlA**  **(13 mice)** | **Utx F2.controlB**  **(19 mice)** | **Utx F2.control(A+B)**  **(32 mice)** |
| --- | --- | --- | --- | --- | --- | --- |
| **All tumors** | 6 (0.24) | 21 (0.95) | 52 (1.30) | 8 (0.62) | 3 (0.16) | 11 (0.34) |
| **All blood tumors** | 2 (0.08) | 8 (0.36) | 9 (0.23) | 2 (0.15) | 0 | 2 (0.06) |
| Histiocytic sarcoma | 1 (0.04) | 6 (0.27) | 6 (0.15) | 1 (0.08) | 0 | 1 (0.03) |
| Lymphoma | 1 (0.04) | 2 (0.09) | 3 (0.08) | 1 (0.08) | 0 | 1 (0.03) |
| **All solid tumors** | 4 (0.16) | 13 (0.59) | 43 (1.08) | 6 (0.46) | 3 (0.16) | 9 (0.28) |
| **Benign solid tumors** | 2 (0.08) | 11 (0.50) | 21 (0.53) | 4 (0.31) | 3 (0.16) | 7 (0.22) |
| Adrenocortical adenoma | 0 | 0 | 2 (0.05) | 0 | 0 | 0 |
| Bronchial papilloma | 0 | 2 (0.09) | 0 | 0 | 1 (0.05) | 1 (0.03) |
| Harderian adenoma | 0 | 1 (0.05) | 1 (0.03) | 1 (0.08) | 0 | 1 (0.03) |
| Hepatocellular adenoma | 2 (0.08) | 2 (0.09) | 7 (0.18) | 2 (0.15) | 2 (0.11) | 4 (0.13) |
| Lung adenoma | 0 | 4 (0.18) | 9 (0.23) | 1 (0.08) | 0 | 1 (0.03) |
| Osteoma | 0 | 1 (0.05) | 1 (0.03) | 0 | 0 | 0 |
| Pancreatic endocrine adenoma | 0 | 0 | 1 (0.03) | 0 | 0 | 0 |
| Pancreatic exocrine adenoma | 0 | 1 (0.05) | 0 | 0 | 0 | 0 |
| **Malignant solid tumors** | 2 (0.08) | 2 (0.09) | 22 (0.55) | 2 (0.15) | 0 | 2 (0.06) |
| Angiosarcoma | 1 (0.04) | 0 | 2 (0.05) | 2 (0.15) | 0 | 2 (0.06) |
| Cholangiocarcinoma | 0 | 0 | 3 (0.08) | 0 | 0 | 0 |
| Colon adenocarcinoma | 0 | 0 | 1 (0.03) | 0 | 0 | 0 |
| Harderian adenocarcinoma | 0 | 0 | 1 (0.03) | 0 | 0 | 0 |
| Hepatocellular carcinoma | 1 (0.04) | 1 (0.05) | 11 (0.28) | 0 | 0 | 0 |
| Lung adenocarcinoma | 0 | 1 (0.05) | 3 (0.08) | 0 | 0 | 0 |
| Rhabdomyosarcoma | 0 | 0 | 1 (0.03) | 0 | 0 | 0 |
